# Phosphorylation of ribosomal protein S6 confers PARP inhibitor resistance in BRCA1-deficient cancers

**DOI:** 10.18632/oncotarget.1952

**Published:** 2014-05-08

**Authors:** Chong-kui Sun, Fan Zhang, Tao Xiang, Qianming Chen, Tej K. Pandita, Yuping Huang, Mickey C.T. Hu, Qin Yang

**Affiliations:** ^1^ Cancer Biology Division, Department of Radiation Oncology, Washington University School of Medicine, Saint Louis, MO 63108; ^2^ State Key Laboratory of Oral Diseases, Sichuan University, Chengdu 610041, China; ^3^ Department of Radiation Oncology, UT Southwestern, Dallas, TX 75390; ^4^ Research Biotechnology Business Unit, Sigma-Aldrich Corporation, St. Louis, MO 63103; ^5^ Division of Gynecologic Oncology, Department of Obstetrics & Gynecology, Stanford University School of Medicine, Stanford, CA 94305

**Keywords:** BRCA1, mTOR, S6, PARP inhibitor, Rapamycin

## Abstract

Inhibition of poly(ADP-ribose) polymerase (PARP) is a promising therapeutic strategy for BRCA1 deficient cancers, however, the development of drug resistance limits clinical efficacy. Previously we found that the BRCA1-AKT1 pathway contributes to tumorigenesis and that the AKT1/mTOR is a novel therapeutic target for BRCA1-deficient cancers. Here, we report that phosphorylation of ribosomal protein S6, a mTOR downstream effector, is greatly increased in BRCA1 deficient cells resistant to PARP inhibition. Phosphorylation of S6 is associated with DNA damage and repair signaling during PARP inhibitor treatment. In BRCA1 deficient cells, expression of S6 lacking all five phosphorylatable sites renders the cells sensitive to PARP inhibitor and increases DNA damage signals. In addition, the S6 mutations reduce tumor formation induced by *Brca1*-deficiency in mice. Inhibition of S6 phosphorylation by rapamycin restores PARP sensitivity to resistant cells. Combined treatment with rapamycin and PARP inhibitor effectively suppresses *BRCA1*-deficient tumor growth in mice. These results provide evidence for a novel mechanism by which BRCA1 deficient cancers acquire drug resistance and suggest a new therapeutic strategy to circumvent resistance.

## INTRODUCTION

The breast cancer susceptibility gene (BRCA1) is commonly found to be mutated in hereditary breast and ovarian cancers. In addition, BRCA1 protein expression is frequently reduced or is absent in sporadic cases, suggesting that it influences both hereditary and sporadic mammary tumorigenesis [1;2]. The BRCA1 protein contains C-terminal tandem BRCT domains that are phosphoprotein binding motifs, which are important for the tumor-suppressor and DNA repair function of BRCA1 [3-5]. Clinically relevant missense mutations identified at the C-terminus of BRCA1 abolish the structure of BRCT. Most *BRCA1* mutations result in truncated *BRCA1* gene products that lack one or both C-terminal BRCT domains [6;7].

BRCA1-deficient cells have compromised DNA repair and are sensitive to poly(ADP-ribose) polymerase (PARP) inhibitors [8-10]. Loss or dysfunction of BRCA1 gene causes a deficiency in the homologous recombination (HR) repair and RAD51 focus formation, which contributes to genomic instability and tumorigenesis [11;12]. PARP inhibition is synthetic lethal with defective DNA repair via HR and, phase 1 as well as phase 2 clinical trials have shown that PARP inhibitors have effective anti-tumor activity for BRCA-associated cancers [13;14]. Despite an initial response, chemo-resistance development eventually limits clinical efficacy [15-18]. The resistant mechanism is unclear, as data from fundamental and preclinical research indicates multiple mechanisms including genetic reversion of BRCA mutations, hypomorphic activity of mutant BRCA1 alleles, upregulation of drug efflux pumps and rewiring of the DNA damage response [19;20]. We previously demonstrated that the AKT/mTOR oncogenic pathway contributes to BRCA1-deficient tumorigenesis and defective DNA repair [21-23]. Here, we identify a novel mechanism for acquired PARP inhibitor resistance by demonstrating that phosphorylation of ribosomal protein S6, a downstream effector of the mTOR pathway, mediates PARP inhibitor resistance through attenuating the DNA damage response and restoring defective HR in BRCA1-deficient cancer cells.

## RESULTS

### Increased phosphorylation of ribosomal protein S6 in BRCA1-deficient cancer cells is associated with resistance to a PARP inhibitor

**Fig. 1:**
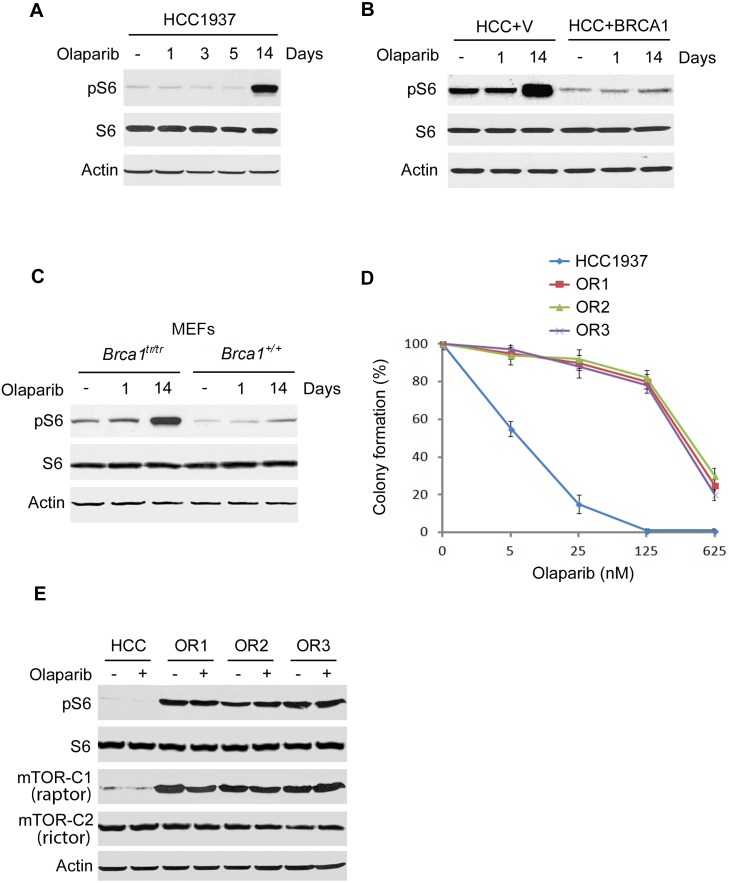
S6 phosphorylation is increased in BRCA1 deficient cells with long time olaparib treatment A. HCC1937 cells (BRCA1-inactive) were treated with 10 nM olaparib with indicated times. Whole-cell lysates were prepared and analyzed by Western blotting with the indicated antibodies. B. Lysates were prepared from that stably expressed BRCA1 or vector only (+v) with or without 10 nM olaparib treatment and analyzed by Western blotting with the indicated antibodies. C. Lysates from *Brca1*^+/+^ or *Brca1*^tr/tr^ MEFs with or without 10 nM olaparib treatment were analyzed by Western blot with the indicated antibodies. D. HCC1937 clones OR1 to OR3 were significantly more resistant to olaparib than parental cells. Colony formation assay was performed (n=3. Mean±SEM of colonies formed relative to DMSO-treated cells). E. Lysates from HCC1937 parental cells and three olaparib resistant clones (OR1 to OR3) were analyzed by Western blot with the indicated antibodies. The cells were treated with 10 nM olaparib for three days.

To examine the role of ribosomal protein S6 in PARP inhibitor resistance, we used a PARP inhibitor olaparib to treat HCC1937 breast cancer cell line. This cell line is hemizygous for the BRCA1 mutant allele 5382insC and therefore expresses a BRCA1 protein lacking the COOH-terminal BRCT repeats. The mutation eliminates the activity of BRCA1 in the repair of DNA damage and maintenance of genomic stability and is associated with an increased risk of cancer. Treatment with olaparib for up to 5 days did not change ribosomal protein S6 phosphorylation in HCC1937 cells (Fig. 1A), however, after two weeks of treatment S6 phosphorylation strongly increased. Expression of wild-type BRCA1 in HCC1937 cells reduced the basal levels of S6 phosphorylation, consistent with our previous reports [22;23] (Fig. 1B). In addition, expression of BRCA1 suppressed the increase in S6 phosphorylation induced by olaparib treatment of two weeks. Furthermore, MEFs expressing a truncated *Brca1* allele (*Brca1^tr/tr^*) [5;22] showed higher S6 phosphorylation level two weeks after olaparib treatment (Fig. 1C), while *Brca1^+/+^* MEFs did not. A similar phenomenon was observed in another BRCA1-mutant cell line SUM149 that S6 phosphorylation level increased after two week treatment with olaparib, but did not change in BRCA1 proficient cell lines MCF7 and MDA-MB-231 (Supplemental Fig. 1 and data not shown). These results suggest that BRCA1 deficiency and S6 phosphorylation are involved in PARP inhibitor resistance.

To verify PARP inhibitor resistance, we cultured HCC1937 in the presence of olaparib and obtained drug-resistant clones emerged 2-3 months after initial exposure. Colony formation assay showed that these clones were highly resistant to olaparib (Fig. 1*D*). Lethal concentrations 50 (LC_50_) were about 450- to 600-fold greater than those for parental cells for olaparib. We measured mTOR and S6 protein levels by Western blot. S6 phosphorylation and its upstream kinase mTORC1 greatly increased in all olaparib resistant clones, whereas the total overall protein levels of S6 and mTORC2 were similar in parental cells and resistant clones (Fig. 1E), indicating that the mTORC1-S6 phosphorylation pathway is involved in PARP inhibitor resistance. To investigate drug efflux as a mechanism of PARP inhibitor resistance, we examined cellular poly(ADP-ribose)(PAR) levels to determine the ability of olaparib to inhibit the PARP enzyme. Western analysis showed that olaparib reduced the levels of PAR to a similar degree in HCC1937 parental cells and in the resistant clones (Supplemental Fig. 2). These results suggest that altered drug efflux does not directly contribute to PARP inhibitor resistance.

### *S6*^P−/−^ HCC1937 cells are sensitive to the PARP inhibitor

**Fig. 2:**
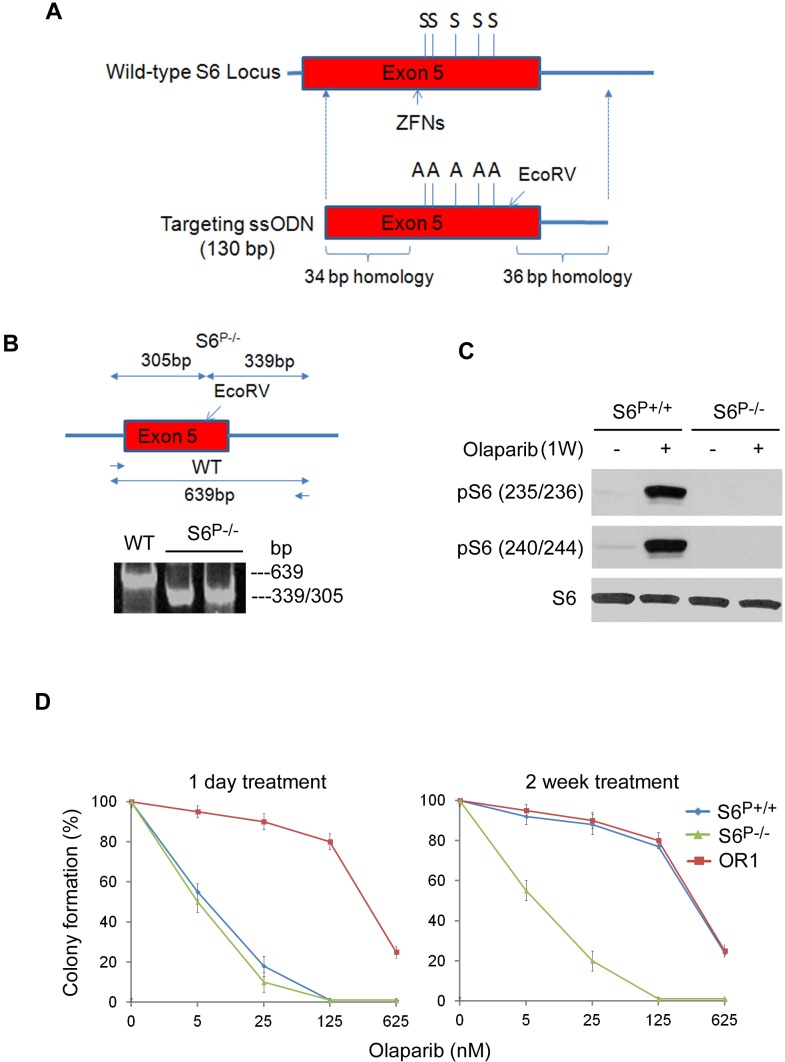
Generation of unphosphorylatable S6 allele (*S6*^P−/−^) in HCC1937 cells A. The structures of endogenous S6 gene, targeting ssODEs. The position of the serine residures (S) and respective alanine substitutes (A) in exon 5 is indicated. B. PCR products of genomic DNA were digested with EcoRV. The wild-type allele (639 bp) and the doublet of the targeted allele (339 and 305 bp) are indicated. C. Lysates from wild-type and *S6*^P−/−^ HCC1937 cells were subjected to Western blot analysis using indicated antibodies. D. *S6*^P−/−^ HCC1937 cells are sensitive to the PARP inhibitor. Colony formation assay was performed after 1 day or 2 weeks 10 nM olaparib treatment in indicated cell types (n=3, mean±SEM of colonies formed relative to DMSO-treated cells). The cells were first selected for the resistance to olaparib (2 weeks treatment) and after that plated for the colony formation assay.

To investigate the role of S6 phosphorylation in PARP inhibitor resistance, we generated unphosphorylatable *S6* knock-in HCC1937 cells. A recent report described a high-frequency genome editing method based on the directional HR mechanism in somatic cells that utilized ssDNA oligonucleotides (ssODNs) with zinc-finger nucleases (ZFN) [24]. Using this method, we replaced all phosphorylatable serine residues (S235, S236, S240, S244, and S247) with alanines in the endogenous *S6* gene (Fig. 2A). We designed a ssODN donor to span the mutation site and the ZFN cleavage site as well as flanking homology sequence. The *S6*-specific ZFNs are fusion proteins including the engineered zinc finger proteins that specifically bind to exon 5 of *S6* genomic DNA, and the non-specific nuclease domain of restriction enzyme FokI that generates double-strand DNA cleavage, which also greatly stimulated HR frequency for ssODN donor replacement. The replacement of genomic DNA with ssODN-S6-130 through the cellular process of HR resulted in creating a knock-in *S6^P−/−^*. To enable restriction fragment length polymorphism (RFLP)-based detection to targeted clones, we also introduced an EcoRV site. After transfection of ssODN S6-130 and mRNA encoding the S6 ZFN into HCC1937 cells, 400 single-cell clones were obtained and screened by RFLP assay (Fig. 2B). Four *S6^P−/−^* monoclonal lines were identified then verified by qPCR, Southern blot, Northern blot and genomic DNA sequencing (Supplementary Fig. 3 and data not shown). Western blot analysis using anti-phospho-Ser^235/236^ or –Ser^240/244^ antibodies, did not detect any phosphorylation of these sites in S6 (Fig. 2C). Together these data demonstrate that these phosphorylatable serine residues in S6 are absent in *S6^P−/−^* HCC1937 cells. To determine the role of S6 phosphorylation in PARP inhibitor resistance, we cultured *S6^P−/−^* HCC1937 cells with olaparib and measured cell survival. Results from colony formation assays indicated that *S6^P−/−^* cells were highly sensitive to olaparib (Fig. 2D) and LC_50_ was about 400- to 600-fold lower than those for olaparib resistance clones. We conclude that S6 phosphorylation may be responsible for PARP inhibitor resistance in BRCA1 deficient cells.

### DNA damage response and repair in *S6*^P−/−^ cells and PARP resistant cells

**Fig. 3:**
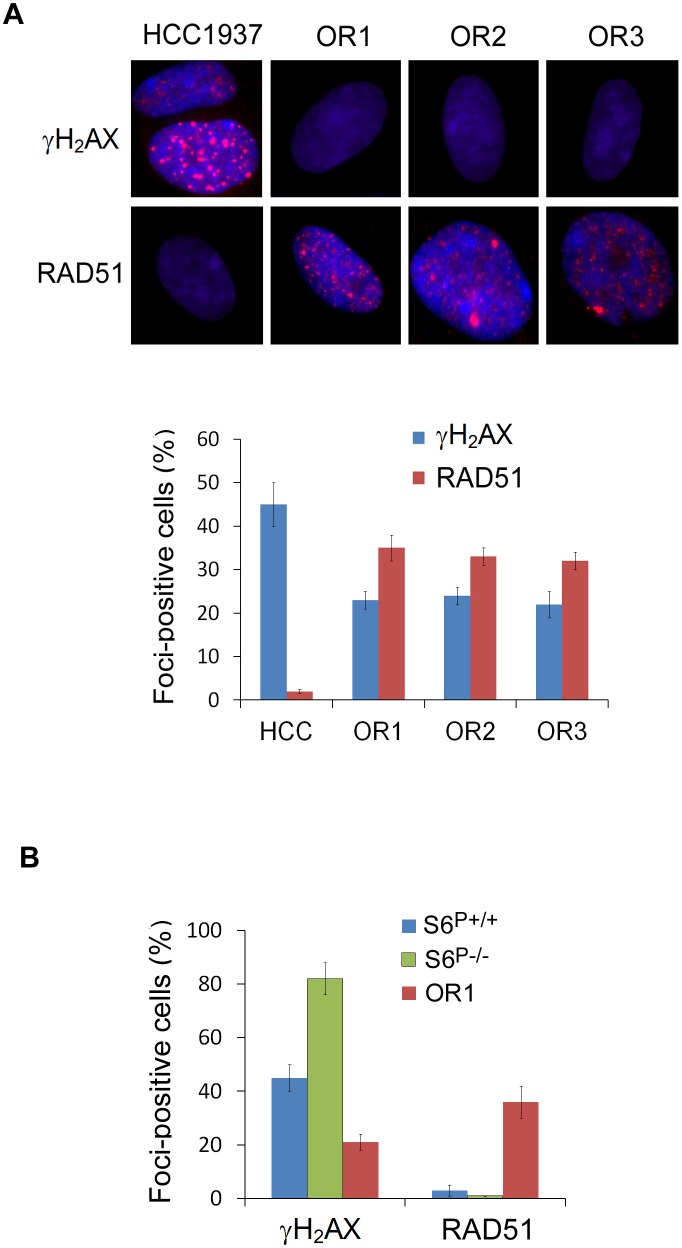
Detection of γH2AX and RAD51 foci in *S6*^P−/−^ cells and PARP resistant cells A. Decrease of γH2AX and increase of RAD51 foci in the PARP resistant HCC1937 clone cells. Immunofluorescence images show γH2AX and RAD51 foci and DAPI counterstain in HCC1937 parental and resistant cells with 2 Gy IR-treatment for 2 h. Upper panel shows representative cells. Bottom panel shows the percentage of cells with γH2AX and RAD51 foci (*n*=200 counted for each cell type). >5 Foci/cell were counted as a positive cell. The *P* values are <0.001 between parental cells and OR clones, based on Student's *t* test. B. Detection of γH2AX and RAD51 foci in *S6*^P−/−^ cells. Quantitative data show the percentage of cells with γH2AX and RAD51 foci (*n*=200). The *P* values are <0.01 among *S6*^+/+^, *S6*^P−/−^ and OR1 cells, based on Student's *t* test.

Next, we examined the DNA damage response by measuring the formation of nuclear foci containing phosphorylated histone γH2AX, a surrogate marker of DNA double-strand break. Immunostaining assay showed that γH2AX foci were readily detected in parental HCC1937 cells following irradiation (IR), consistent with the previous report [21]. In the olaparib resistant clones, γH2AX foci were significantly decreased, compared with those in the parental cells (Fig. 3A; Supplementary Fig. 4). Furthermore, quantitative data revealed a 2-fold increase in *S6^P−/−^* HCC1937 cells with H2AX foci, compared with *S6* wild-type cells (Fig. 3B; Supplementary Fig. 5). Thus, these data indicate that S6 phosphorylation is involved in the DNA damage response.

BRCA1-deficient cells display defective in HR [11;12]. Therefore, we measured RAD51 focus formation after IR, a marker for HR, which is impaired in BRCA1-deficient cells [25-27]. Wild-type, PARP resistant clone and *S6^P−/−^* HCC1937 cells were treated with IR and stained with a RAD51 antibody (Fig. 3B, Supplementary Fig. 5). Analysis of these IR-induced RAD51 foci revealed a diminished response upon depletion of BRCA1, consistent with previous observations [25;27]. PARP resistant clones readily formed RAD51 foci following IR. Moreover, RAD51 foci could not be detected in *S6^P−/−^* HCC1937 cells. These data suggest that S6 phosphorylation might regulate the HR process.

### Rapamycin restores sensitivity of HCC1937 resistant clones to the PARP inhibitor

**Fig. 4:**
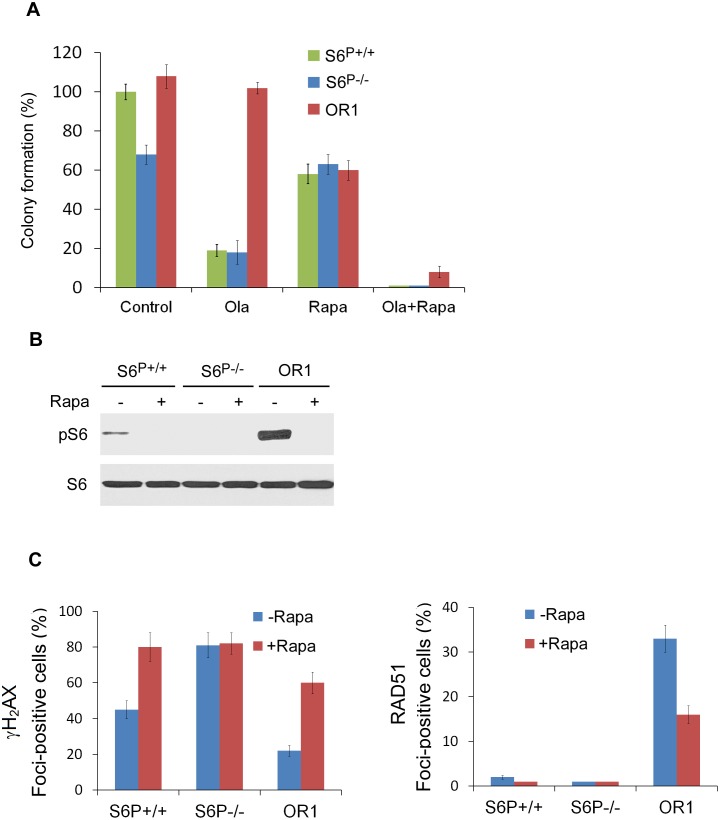
Rapamycin restores sensitivity of HCC1937 resistant clones to the PARP inhibitor A. Colony formation assay was performed in HCC1937 parental and olaparib resistant cells, and *S6*^P−/−^ cells with or without 1 µM rapamycin and/or 10 nM olaparib treatment (n=3, mean±SEM of colonies formed relative to DMSO-treated cells). B. Whole-cell lysates were analyzed by Western blotting with the indicated antibodies. C. Detection of γH2AX and RAD51 foci. Quantitative data show the percentage of cells with γH2AX and RAD51 foci (*n*=200). >5 Foci/cell were counted as a positive cell. The *P* values are <0.01 between treatment with and without rapamycin in *S6*^+/+^ and OR1 cells, based on Student's *t* test.

To further study the role of mTOR/S6 pathway in PARP resistance, we tested the effect of rapamycin, a clinically used selective inhibitor of mTORC1 and S6 phosphorylation. Treatment with rapamycin alone produced a reduction of colony formation in the similar level in HCC1937 *S6^P−/−^*, olaparib resistant and parental cells (Fig. 4A; Supplementary Fig. 6). Furthermore, these cells were very sensitive to combinational treatments of rapamycin with olaparib, and almost no clones were formed, suggesting that rapamycin could overcome olaparib resistance and S6 phosphorylation may be associated with it. Western blot analysis showed that rapamycin treatment suppressed S6 phosphorylation in olaparib resistant cells as well as in HCC1937 parental cells (Fig. 4B), supporting a role for S6 phosphorylation in PARP inhibitor resistance. As expected, rapamycin treatment significantly increased γH2AX foci and decreased RAD51 foci in the olaparib resistant clone (Fig. 4C). Taken together, these data indicate that rapamycin may restore BRCA1 deficient cell sensitivity to olaparib through inhibition of S6 phosphorylation.

### Combined rapamycin and PARP inhibitor treatment effectively suppresses *BRCA1*-deficient tumor growth in mice

**Fig. 5:**
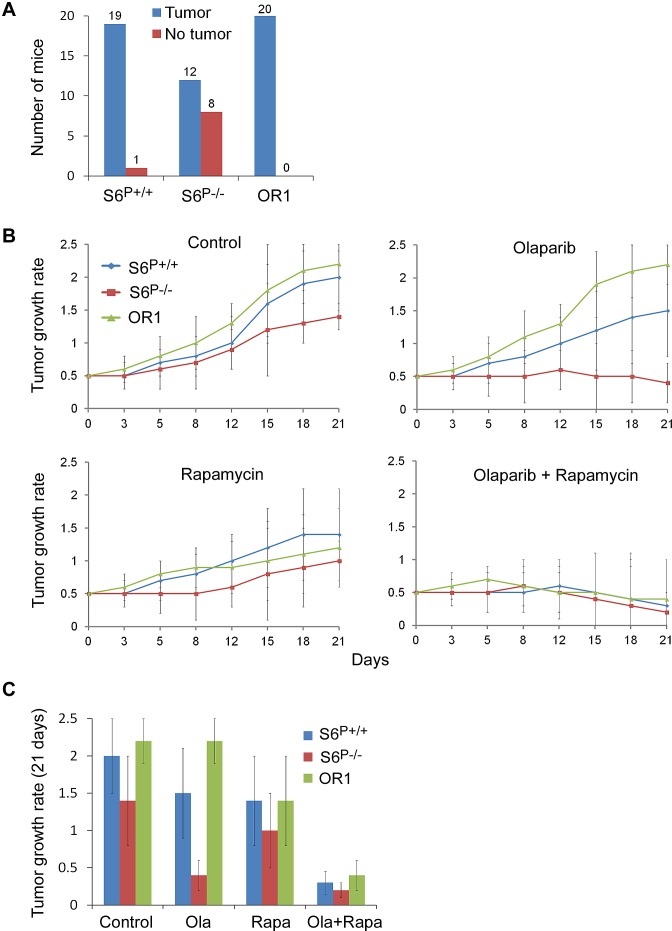
Combinational treatment of rapamycin with olaparib effectively suppresses *BRCA1*-deficient tumor growth in mice A. *S6*^P−/−^ HCC1937 cells-induced tumors in mice are decreased. After 9 weeks, the tumors became rigid and the volume of tumor ((L × W^2^)/2) is measured (n=20 each group). The *P* values are <0.01 between *S6*^+/+^/OR1 and *S6*^P−/−^ groups, based on Student's *t* test. B. Combinational treatment of rapamycin with olaparib significantly suppresses tumor growth in the xenograft tumors. Tumor growth rates after the drug treatments are shown (*S6*^+/+^, n=19; *S6*^P−/−^, n=12; OR1, n=20). The mice bearing xenograft tumors were assigned to vehicle and treatment groups and mean tumor volumes at the start point (after 9 weeks implanting cells) for treatment were indistinguishable between the vehicle and treatment groups. C. Statistical analysis of effects of the drugs on tumor growth for mice bearing xenograft tumors (after 18 days treatment). The *P* values are <0.001 between olaparib alone and rapamycin+olaparib treatment in *S6*^+/+^and OR1 groups, and between parental control and *S6*^P−/−^ tumors with olaparib alone treatment, based on Student's *t* test.

The colony formation assay showed reduced cell viabilities in *S6^P−/−^* HCC1937 cells compared with parental control cells (Fig. 4A), suggesting that S6 phosphorylation may be involved in tumorigenesis. Therefore, HCC1937 *S6^P−/−^*, olaparib resistant and parental cells were implanted into SCID mice and tumor formation was monitored for 9 weeks. Implantation of control parental HCC1937 cells resulted in tumor formation in 19 of 20 mice (Fig. 5A). Removal of the S6 phosphorylations was sufficient to suppress tumor development and only 12 of 20 mice generated tumors. All olaparib resistant cells generated tumors (n=20). In addition, these resistant cells also resulted in larger tumors compared with those from parental control cells (Fig. 5 and data not shown).

**Fig. 6:**
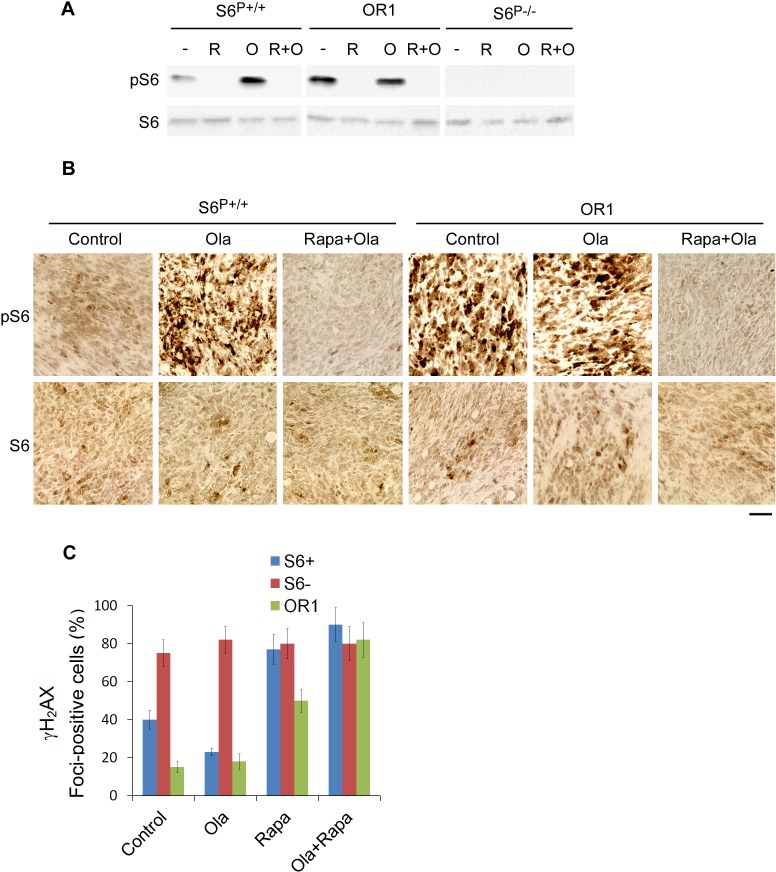
Rapamycin treatment inhibits the pS6 expression level in the xenograft tumors in mice A. Western blot analysis was performed from the xenograft tumors in mice with indicated antibodies. B. IHC analysis of pS6 and S6 in the xenograft tumors with or without indicated drug treatment. Original magnification × 200. Scale bar, 10 µm. C. Detection of γH2AX foci in the xenograft tumors. Quantitative data show the percentage of cells with γH2AX foci (*n*=200). >5 foci/cell were counted as a positive cell.

We tested the effects of rapamycin and olaparib on *Brca1*-deficient tumors in mice and found that olaparib significantly inhibited *S6^−/−^* tumor growth. Rapamycin treatment inhibited tumor growth from all transplanted cell lines including olaparib resistant cells (Figs. 5BC), suggesting that rapamycin may have better therapeutic effects on olaparib resistant tumors and the tumors with high expressing level of S6 phosphorylation. Consistent with this hypothesis, combination treatments of rapamycin with olaparib had a more inhibitory effect for olaparib resistant cell-induced tumors as well as parental cell-induced tumors (Figs. 5BC). Analyses of tumor lysates by Western blot and IHC assays indicated that rapamycin greatly reduced the levels of S6 phosphorylation (Figs. 6AB), consistent with rapamycin inhibition of the mTOR signaling. Consistent with *in vitro* data, rapamycin treatment significantly increased γH2AX foci in the olaparib resistant cell-induced tumors and parental control cell-induced tumors (Fig. 6C).

## DISCUSSION

Previously we found that the BRCA1-AKT1 pathway contributes to tumorigenesis and that the AKT1/mTOR is a novel therapeutic target for BRCA1-deficient cancers [21-23]. Here, we provide evidence that, under PARP inhibitor selection pressure, phosphorylation of ribosomal protein S6 is dramatically increased. Using both rapamycin and a S6 mutant deficient for phosphorylation, we have shown that S6 phosphorylation is an important mediator of the PARP inhibitor resistance. We have found a synergy between rapamycin and olaparib for the treatment of BRCA1-mutant and olaparib resistant tumors. Importantly, S6 phosphorylation attenuates DNA damage and promotes RAD51 loading onto DNA following DNA damage. Both rapamycin and the phosphorylation deficient S6 mutant enhance γH2AX focus formation without altering PAR activity, suggesting increased DNA damage when S6 phosphorylation is inhibited in the context of BRCA1 mutation. *In vivo* γH2AX foci in tumors were increased when mice were treated with rapamycin during the period of response, supporting a progressive accumulation of unrepaired DNA DSBs. Consistent with this notion, a recent report has shown that S6 phosphorylation attenuates DNA damage and tumor suppression in pancreatic cancer [28]. Of particular interest is our observation that inhibition of S6 phosphorylation greatly reduces RAD51 focus formation in the PARP resistant cells. These results suggest that S6 phosphorylation is required for recruitment of RAD51 into DNA damage sites. Because BRCA1 is a key factor recruiting RAD51 to DNA damage sites, it is possible that S6 phosphorylation becomes more critical for this recruitment in BRCA1 deficient cells. Previous reports have shown that the PTEN-PI3K pathway contributes to DNA DSB accumulation [29-31], possible through regulation of PARP activation. It would not contribute to the reliance on PARP activity for DNA damage repair because we do not observe alteration of PARP activity in our model. In future studies, additional experiments will be needed to understand the molecular mechanism of these factors in DNA repair processes.

Recent reports show that 53BP1 loss rescues *BRCA1* deficiency and is associated with triple-negative and *Brca1*-mutated breast cancer [32;33]. However, other reports indicate that 53BP1 depletion only partly contributes to PARP inhibitor resistance [34] and confers only a slight degree of resistance [15;35]. 53BP1 inhibits HR in *Brca1*-deficient cells by blocking resection of DNA breaks. We have previously shown that the AKT pathway is associated with HR in *BRCA1*-deficient cells [21]—this is somewhat similar to the ability of 53BP1 loss rescuing HR in *BRCA1*-deficient cells. We did not observe 53BP1 changes during olaparib treatment in our model (Supplementary Fig. 7), suggesting that S6 phosphorylation and 53BP1 independently regulate HR in *BRCA1*-deficient cells resistant to PARP inhibitor. 53BP1 and BRCA1 may regulate the choice between HR and non-homologous end joining. One critical function for BRCA1 might be to remove end-joining proteins such as 53BP1 from replication-associated breaks. Thus, BRCA1 might regulate phosphorylated S6 and 53BP1 in different steps during the HR process. The molecular details of these proteins in the HR process need further investigation in the future.

One mechanism of PARP resistance is upregulation of drug efflux pumps [19;20]. Although we did not observe any change in PARP activity in resistant cells, we cannot rule out that S6 phosphorylation might indirectly contribute to PARP inhibition resistance through this mechanism. PARP activation induces the depletion of its substrate NAD^+^ and consecutively depletion of ATP, resulting in energy loss and eventually cell apoptosis. Previous reports have shown that S6 phosphorylation is a determinant of glucose homeostasis [36;37]. *S6*^P−/−^ mice have impaired glucose tolerance through insulin deficiency [37], leading to decreased energy production via glycolysis. Both glycolysis and PAR consume NAD^+^ and compete for NAD^+^ in the cytosol. PARP inhibition spares NAD^+^ and generates a prosurvival effect for PARP inhibitor resistant cells. Inhibition of S6 phosphorylation leads to low level of glucose supply and glycolytic activity, leading to cell apoptosis. Therefore, a possible interpretation for the synergy of S6 phosphorylation and PARP inhibition is that inhibition of S6 phosphorylation reverses the effects of PARP inhibitor resistance on cell survival. Consistent with this model, a recent report shows that PI3K inhibition can reverse response of PARP inhibition through enhancement of glucose uptake and AKT phosphorylation [31].

Collectively, S6 phosphorylation might play a key role in PARP inhibitor resistance through regulations of DNA damage response/the HR repair process and glucose metabolism. Combinational inhibitions of S6 phosphorylation and PARP might predict to be particularly effective in cancers with PARP inhibitor resistance and HR defects, such as BRCA1-deficient breast and ovarian cancers.

## MATERIALS AND METHODS

### Cell culture, lentivirus infection and western blot analysis

HCC1937, *Brca1*^+/+^ and *Brca1^tr/tr^* MEFs, SUM149, MCF7 and MDA-MB-232 breast cancer cells were cultured as described previously [21-23]. To generate lentiviral particles, 293 T cells were cotransfected with the lentiviral vectors and compatible packaging plasmids mixture using Lipofectamine 2000 (Invitrogen, Carlsbad, CA, USA), and the lentivirus supernatant was collected 40 h after transfection. For virus infection, cells were exposed to lentivirus supernatant for 24 h in the presence of polybrene (Sigma, St Louis, MO, USA). Protein extracts from cells and xenograft tumors were extracted to conduct western blot analysis as described previously [23;38-40].

### Antibodies and reagents

Anti-S6 ribosomal protein antibodies S6 and pS6, anti-mTOR (raptor and rictor) and anti-PARP antibodies were from Cell Signaling Technology. Anti-β-actin antibody was from Sigma. The goat anti-mouse IgG-HRP, goat anti-rabbit IgG-HRP, goat anti-mouse IgG-biotin and goat anti rabbit-IgG-biotin second antibodies were from Santa Cruz Biotechnology (Santa Cruz, CA, USA). Vectastain ABC kit and DAB substrate kit were from Vector Laboratories.

### Knock-in of *S6^P−/−^* by zinc finger nucleases (ZFNs) in HCC1937 cells

CompoZr Knockout Zinc Finger Nucleases targeting human *S6* exon 5 genomic DNA were from Sigma. *S6* ZFN-mRNAs were transfected into HCC1937 cells and Cel-I assay was performed to check efficiency of depleting *S6*. ssODN-S6-130 was designed to replace all phosphorylatable serine residues 235, 236, 240, 244, and 247 with alanines in the *S6* gene with an EcoRV site. ssODN-S6-130 was synthesized and co-transfected with *S6* ZFN-mRNAs into HCC 1937 cells. The cells were plated in100mm dish for single clone formation. The 400 single cell clones were picked, cultured and screened by RFLP assay. Four *S6^P−/−^* clones were verified by qPCR, Southern, Northern, Western blot analysis and genomic DNA sequence.

### Indirect immunofluorescence

Experiments were performed as described [39;41;42]. Briefly, for conventional immunofluorescence microscopy, the cells were examined with a Zeiss Axioskop fluorescence microscope equipped with a CCD camera (Ontario, NY, USA). Images were captured, pseudocolored and merged using IPLab image analysis software. At least 200 cells were analyzed for each experiment. The experiments were repeated at least three times.

### Colony formation assays

Cells were plated and cultured in 60 mm dishes in triplicate. Cells were incubated for about 14–16 days until colonies were large enough to visualize. Colonies were observed under phase contrast microscope and viable cell clones were measured. All data were normalized relative to the control. Experiments were performed three times.

### Tumor growth and treatment experiments

Animal experiments were performed according to institutional guidelines for animal welfare. Female NOD.SCID/NCR mice of 6–8 weeks of age were purchased from NCI-Frederick Animal Production Program (Frederick, MA, USA). In all, 2 × 10^6^ HCC1937 *S6^P−/−^*, olaparib resistant and parental cells in 0.1 ml PBS were mixed with equal volume of matrigel. The cell mixture was implanted into mouse breast fat pad. After 9 weeks, the tumors became rigid and the volume of tumor ((L × W^2^)/2) is measured in range of 180–250 mm^3^. The mice were assigned to vehicle and treatment groups and mean tumor volumes at the start point for treatment were indistinguishable between the vehicle and treatment groups. Rapamycin was injected at 0.3 mg/kg diluted in 100 μl of vehicle on the first treatment and then at 0.15 mg/kg every other day for 18 days. Olaparib was used at 50 mg/kg every other day for 18 days. Control mice received 100 μl of vehicle only. At the end of treatment, the mice were killed and the tumors were excised and processed to paraffin section and protein extraction.

### IHC assay

IHC was performed by using S6 and pS6 antibodies. Paraffin slides were deparaffinized and rehydrated by sequential incubations in xylene, 100% ethanol and 95% ethanol. Endogenous peroxidases were quenched for 20 min with 3% H_2_O_2_ at room temperature. An antigen retrieval step was performed by placing slides in preheated sodium citrate buffer (10 mM, pH 6.0) and heated for 10 min in a pressure cooker. The slides were allowed to cool to room temperature. Slides were blocked with 5% of goat serum in Tris buffered saline (TBS) buffer for 60 min at room temperature. The diluted primary antibody was then added and incubated in a moist chamber at 4 °C overnight. Biotinylated secondary antibody was added for 30 min at room temperature. To detect primary antibody binding, ABC and DAB kits were applied according to the manufacturer instruction (Vector Laboratories). After mounting, the slides were observed under microscope and pictures were taken.

## SUPPLEMENTARY TABLES


