# Supplementary material for: Phosphorylation of ribosomal protein S6 confers PARP inhibitor resistance in BRCA1-deficient cancers
Source: Oncotarget. 2014 May 8;5(10):3375–85. doi: 10.18632/oncotarget.1952 (PMC4102816; doi:10.18632/oncotarget.1952)
Supplement: Supplementary file 1 [file oncotarget-05-3375-s001.pdf]

Phosphorylation of ribosomal protein S6 confers PARP inhibitor resistance in BRCA1-deficient cancers

Supplemental Information

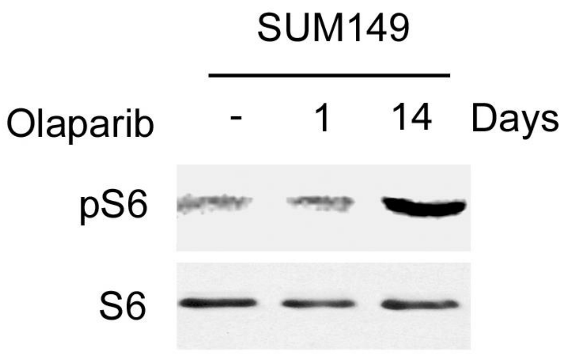

S-Fig. 1: S6 phosphorylation is increased in BRCA1 deficient cells with 14-day olaparib treatment. SUM149 cells (BRCA1-inactive) were treated with 10 nM olaparib with indicated times. Whole-cell lysates were prepared and analyzed by Western blotting with the indicated antibodies.

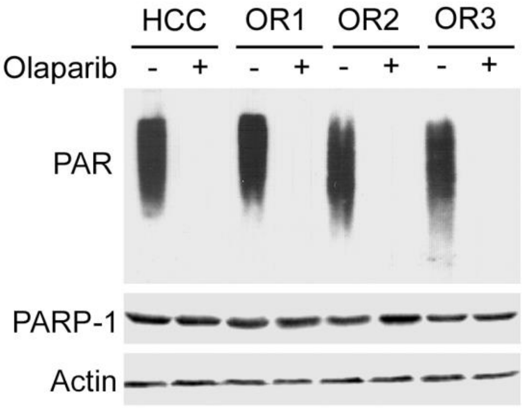

S-Fig. 2: Measurement of PAR levels and PARP activity. HCC1937 parental cells and resistant clones were treated with 1 µM olaparib for 24 h. PAR levels and PARP activity were measured in cell lysates by Western blot with indicated antibody in the absence of activated DNA.

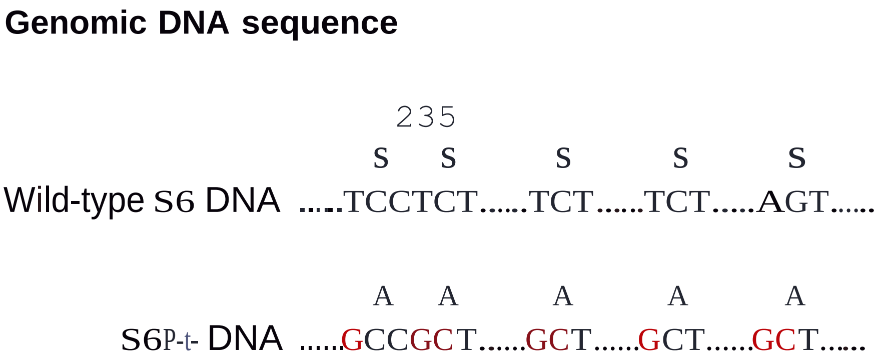

S-Fig. 3: Knock-in of *S6* $^{P-/-}$ in HCC1937 cells by ZFNs. Exon 5 of *S6* gene wild-type and *S6* $^{P-/-}$ sequencing results.

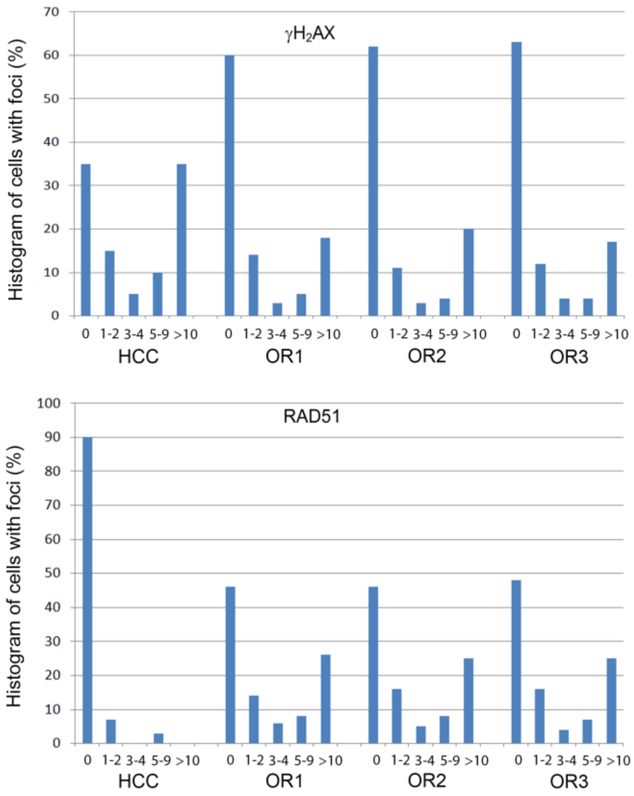

S-Fig. 4: Decrease of γH2AX and increase of RAD51 foci in PARP resistant cells. Immunofluorescence images show γH2AX and RAD51 foci in HCC1937 parental and resistant cells with 2 Gy IR-treatment for 2 h. Quantitative data show the histogram of cells with γH2AX foci (*n*=200).

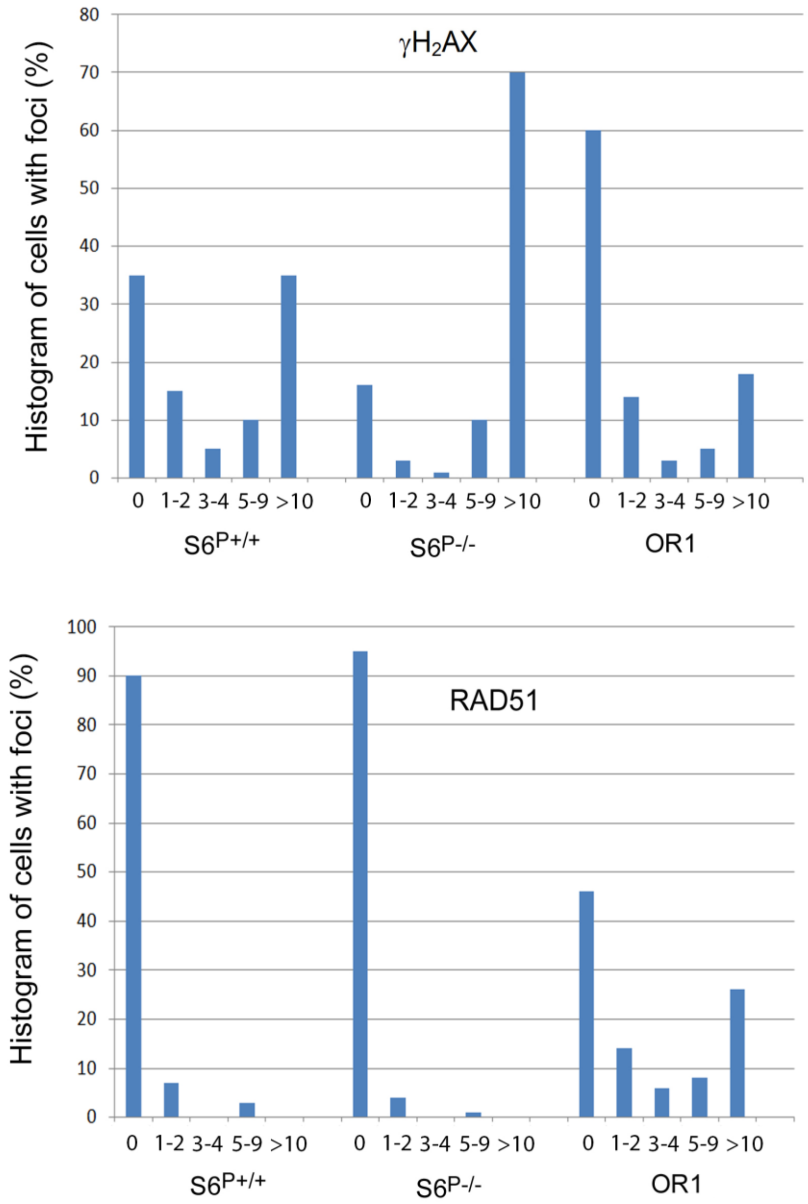

S-Fig. 5: Detection of γH2AX and RAD51 foci in *$S6^{P-/-}$* cells and PARP resistant cells.

Quantitative data show the histogram of cells with γH2AX and RAD51 foci (*n*=200)

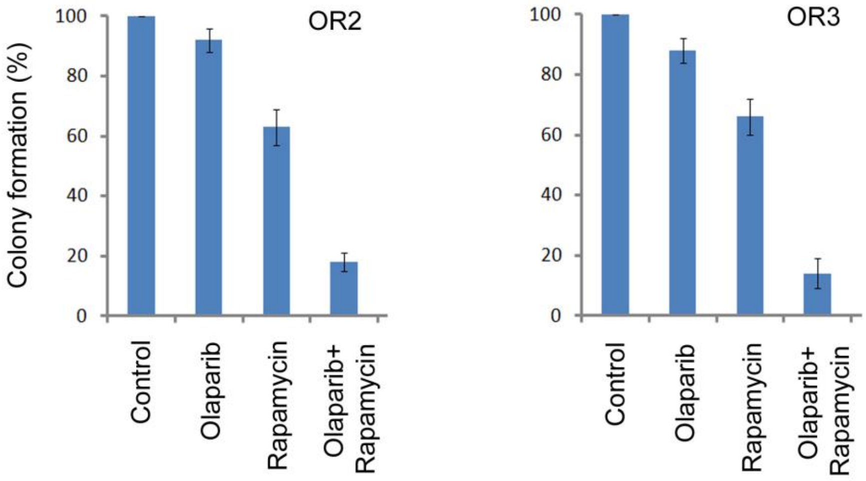

S-Fig. 6: Rapamycin restores sensitivity of HCC1937 resistant clones to the PARP inhibitor. Colony formation assay was performed in HCC1937 parental and olaparib resistant clone cells (OR2 and OR3) with 1 µM rapamycin and/or 10 nM olaparib treatment (n=3, mean±SEM of colonies formed relative to DMSO-treated cells).

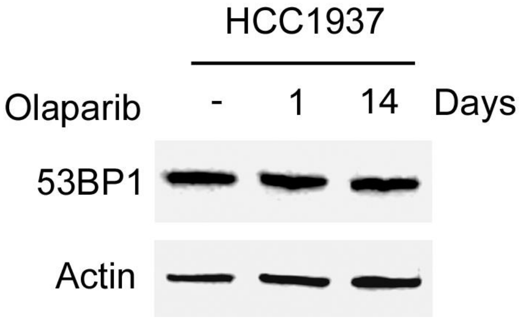

S-Fig. 7: Olaparib treatment does not change the level of 53BP1 protein in BRCA1 deficient cells. HCC1937 cells were treated with 10 nM olaparib with indicated times. Whole-cell lysates were prepared and analyzed by Western blotting with the indicated antibodies.
